# Introduction to Special Issue of Radiology and Imaging of Cancer

**DOI:** 10.3390/cancers12092665

**Published:** 2020-09-18

**Authors:** Roberta Fusco, Vincenza Granata, Antonella Petrillo

**Affiliations:** Radiology Division, Istituto Nazionale Tumori—IRCCS—Fondazione G. Pascale, Via Mariano Semmola, 80131 Naples, Italy; r.fusco@istitutotumori.na.it (R.F.); a.petrillo@istitutotumori.na.it (A.P.)

**Keywords:** MRI, CT, PET, oncology, computed analysis

The increase in knowledge in oncology and the possibility of creating personalized medicine by selecting a more appropriate therapy related to the different tumor subtypes, as well as the management of patients with cancer within a multidisciplinary team has improved the clinical outcomes [1]. 

The characteristics involved are a more appropriate surveillance for the patient at risk to obtain an early diagnosis of the disease, an improvement in the efficacy of therapies based on better patient selection [1,2] and, thanks to a more strategic approach, the possibility of identifying responders or non-responders to therapies as soon as possible, not least the possibility of selecting different treatments related to genomic data. In fact, it has been shown that genomic markers such as microRNA expression are associated with the response to treatment, metastatic spread and prognosis that could offer personalized and precision medicine. In this scenario, the radiologist’s rule has profoundly changed.

Medical imaging comprises a huge number of imaging techniques, and multiple biomedical imaging techniques are used in all phases of cancer management because they are able to provide morphological and functional data [2,3,4,5,6,7,8,9,10,11,12,13,14,15,16,17,18,19]. In addition, image-guided invasive therapy has the promise to improve outcomes and reduce collateral effects compared to systemic or surgical treatment [20,21]. Hybrid imaging techniques are able to supply complementary information for improved staging and therapy planning [22,23,24]. Early detection of cancer through screening based on imaging is probably the major contributor to a reduction in mortality for certain cancers [25]. Nowadays, imaging can also characterize several lesions and predict their histopathological features and can predict tumor behavior and prognosis [26]. 

Imaging diagnosis can be assisted by computed analysis. At present, new parameters can be evaluated using computer-assisted imaging analysis that would be not visible during standard radiological evaluation and reporting and can be used for oncologic evaluations and response to therapy and could cover new significant roles, such as the evaluation of tumor aggressiveness and prognostic prediction.

Recent technological advances in medical imaging, especially in the field of artificial intelligence for the processing of large quantities of iconographic data from different imaging modalities, hold promise in addressing clinical challenges in cancer detection, assessment of treatment response, and monitoring disease progression [10,12,13,27,28,29].

In this context three different broad approaches can be identified, whose tentative definitions, although continuously updated, can be currently outlined as radiomics, machine learning and deep learning. 

Radiomics includes feature extraction from clinical images; these features are related to tumor size, shape, intensity, and texture, collectively providing comprehensive tumor characterization (the so-called radiomics signature of the tumor) [30,31]. The use of imaging data from routine clinical work-up has tremendous potential in improving cancer care by heightening understanding of tumor biology and aiding in the implementation of precision medicine. As a noninvasive method of assessing the tumor and its microenvironment in their entirety, radiomics allows the evaluation and monitoring of tumor characteristics such as temporal and spatial heterogeneity [32].

Machine learning (ML) is a branch of artificial intelligence centered on algorithms which do not need explicit prior programming to function but automatically learn from available data, creating decision models to complete tasks. ML-based tools have been proposed for applications in oncology for oncological risk assessment, automated segmentation, lesion detection, characterization, grading and staging, prediction of prognosis and therapy response [33].

Deep learning is a renewed area of research that deals with development of deep artificial neural networks that were inspired by biological neural networks in our brain. In radiology, deep neural networks, like biological neural networks, attempt to learn an intrinsic representation of the radiological data. Deep learning has become an active area of research in the field of computer-assisted clinical and radiological decision support in recent years, with some excellent initial results that have been recently surveyed [27,34].

Technological developments will increase imaging speed to match that of physiological processes. Targeted imaging and therapeutic agents will be developed in tandem through close collaboration between academia and biotechnology, information technology and pharmaceutical industries.

The aim of this Special Issue is to present new challenges in cancer imaging, including the potential applications of radiomics and artificial intelligence in several malignancies.
